# Transmission pathways of *Campylobacter jejuni* between humans and livestock in rural Ethiopia are highly complex and interdependent

**DOI:** 10.1186/s13099-025-00691-7

**Published:** 2025-05-03

**Authors:** Nitya Singh, Cecilie A. N. Thystrup, Bahar Mummed Hassen, Menuka Bhandari, Gireesh Rajashekara, Tine M. Hald, Mark J. Manary, Sarah L. McKune, Jemal Yusuf Hassen, Helen L. Smith, Jonathan C. Marshall, Nigel P. French, Arie H. Havelaar, The CAGED Research Team Members consisted of, Abadir Jemal Seran, Abdulmuen Mohammed Ibrahim, Amanda E. Ojeda, Bahar Mummed Hassen, Belisa Usmael Ahmedo, Cyrus Saleem, Dehao Chen, Efrah Ali Yusuf, Getnet Yimer, Ibsa Abdusemed Ahmed, Ibsa Aliyi Usmane, Jafer Kedir Amin, Kedir Abdi Hassen, Kedir Teji Roba, Kunuza Adem Umer, Karah Mechlowitz, Loic Deblais, Mahammad Mahammad Usmail, Mark J. Manary, Mawardi M. Dawid, Mussie Bhrane, Nur Shaikh, Wondwossen Gebreyes, Xiaolong Li, Yang Yang, Yenenesh Demisie Weldesenbet, Zelalem Hailu Mekuria

**Affiliations:** 1 Department of Animal Sciences, Global Food Systems Institute, Emerging Pathogens Institute, University of Florida, Gainesville, FL USA; 2 National Food Institute, Technical University of Denmark, Kgs. Lyngby, Denmark; 3 College of Veterinary Medicine, Haramaya University, Haramaya, Oromia Regional State Ethiopia; 4 Department of Biomedical Sciences and Pathobiology, VA-MD College of Veterinary Medicine, Virginia Tech, Blacksburg, VA USA; 5 Department of Pathobiology, College of Veterinary Medicine, University of Illinois, Urbana, IL USA; 6 Department of Pediatrics, Washington University, St. Louis, MO USA; 7 Department of Environmental and Global Health, Center for African Studies, University of Florida, Gainesville, FL USA; 8 Office of Research Affairs, Haramaya University, Haramaya, Oromia Regional State, Ethiopia; 9 School of Mathematical and Computational Sciences, Massey University, Palmerston North, New Zealand; 10 School of Veterinary Science, Massey University, Palmerston North, New Zealand

**Keywords:** *Campylobacter jejuni*, Attribution, Transmission pathways, Zoonosis, Diversity, Persistence, Spatial distribution, Sequencing typing

## Abstract

**Background:**

*Campylobacter jejuni* and *C. coli* are the most common causes of bacterial enteritis worldwide whereas symptomatic and asymptomatic infections are associated with stunting in children in low- and middle-income countries. Little is known about their sources and transmission pathways in low- and middle-income countries, and particularly for infants and young children. We assessed the genomic diversity of *C. jejuni* in Eastern Ethiopia to determine the attribution of infections in infants under 1 year of age to livestock (chickens, cattle, goats and sheep) and other humans (siblings, mothers).

**Results:**

Among 287 *C. jejuni* isolates, 48 seven-gene sequence types (STs), including 11 previously unreported STs were identified. Within an ST, the core genome STs of multiple isolates differed in fewer than five alleles. Many of these isolates do not belong to the most common STs reported in high-resource settings, and of the six most common global STs, only ST50 was found in our study area. Isolates from the same infant sample were closely related, while those from consecutive infant samples often displayed different STs, suggesting rapid clearance and new infection. Four different attribution models using different genomic profiling methods, assumptions and estimation methods predicted that chickens are the primary reservoir for infant infections. Infections from chickens are transmitted with or without other humans (mothers, siblings) as intermediate sources. Model predictions differed in terms of the relative importance of cattle versus small ruminants as additional sources.

**Conclusions:**

The transmission pathways of *C. jejuni* in our study area are highly complex and interdependent. While chickens are the most important reservoir of *C. jejuni*, ruminant reservoirs also contribute to the infections. The currently nonculturable species *Candidatus* C. infans is also highly prevalent in infants and is likely anthroponotic. Efforts to reduce the colonization of infants with *Campylobacter* and ultimately stunting in low-resource settings are best aimed at protecting proximate sources such as caretakers’ hands, food and indoor soil through tight integration of the currently siloed domains of nutrition, food safety and water, sanitation and hygiene.

**Supplementary Information:**

The online version contains supplementary material available at 10.1186/s13099-025-00691-7.

## Background

*Campylobacter jejuni* and *C. coli* are the most common causes of bacterial enteritis worldwide. The World Health Organization estimates these bacteria cause 166 million cases of gastroenteritis annually [1]. The highest incidence rate is observed in low- and middle-income countries, especially in Africa (2180 cases per 100,000 persons per year (ppy) versus 350 cases per 100,000 ppy in North America). Approximately 60% of these cases are foodborne. Other transmission patterns include animal contact (approx. 15%), water (approx. 10%), human contact, soil and other unspecified pathways (approx. 5% each) [2]. Moreover, the Malnutrition and Enteric Disease (MAL-ED) study has suggested that symptomatic and asymptomatic infections with several enteric pathogens including *C. jejuni* and *C. coli* are associated with stunting in children in low- and middle-income countries [3]. The vaccination of rhesus macaques against *C. coli* not only reduced the incidence of overt diarrhea but also improved their linear growth [4].

We previously reported a high prevalence of bacteria of the *Campylobacter* genus in humans and livestock in smallholder households in Haramaya woreda, East Hararghe Zone, Oromia State, Ethiopia [5]. The prevalence and load in infants increased significantly with age with logistic regression predicting a prevalence of approximately 90% at 1 year of age. Most infections are asymptomatic, but the bacterial load is positively correlated with the risk of diarrhea [6]. The load of *Campylobacter* in infant stools was greater in girls than in boys and increased with increasing food insecurity, different feeding practices (prelacteal feeding, early introduction of complementary foods, consumption of any solid foods, drinking of raw milk, household ownership of cattle and sheep but not chickens or goats), hygiene-related factors (improper disposal of infant stools, contact with animals or their feces, mouthing soil) and treatment with antibiotics in the previous month. Mothers’ handwashing with soap and drinking from bottles with nipples were associated with lower loads [6, 7]. Two main species were found in infants by real-time Polymerase Chain Reaction (qPCR) and shotgun metagenomic sequencing: *Candidatus* C. infans (*C. infans*, 60% at 1 year of age) and *C. jejuni* (50% at 1 year of age). *C. upsaliensis* was also detected but less frequently (20% at 1 year of age). *C. coli* was not detected in infants by shotgun sequencing and was detected infrequently (1%) by qPCR [8, 9].

In high-income countries, animals, specifically livestock, are considered the primary reservoirs of human infections with the well-studied species *C. jejuni/coli*, with foodborne transmission [2], mainly through poultry meat [2] as the main pathway. However, less is known about their sources and transmission pathways in low- and middle-income countries, particularly for infants and young children.

The load of *C. infans* was greater in girls than in boys and was also elevated for infants who drank raw milk or crawled in areas contaminated with animal feces, whereas the load of *C. jejuni* was greater for infants who put soil in their mouths. At both the genus and species level, there were mixed and often counterintuitive signals related to keeping animals in the home, whether confined or not [6, 8]. These results suggest a complex contamination network among humans, animals and their environment.

Source attribution of *C. jejuni/coli* has largely been based on legacy (seven-gene) Multi Locus Sequence Typing (MLST) [10], using frequency matching models such as the Dutch model [11], Hald model [12] and variants [13], or population genetic models such as the asymmetric Island model [14] or STRUCTURE [15]. More recently, attribution models based on whole-genome sequencing (WGS) data have been developed. WGS data enhance the ability to distinguish genetic variations and potentially more accurately determine the origin of infection-causing isolates [16]. Both core genome MLST (cgST) and *k*-merization have been used for taxonomic profiling and are particularly effective for large genomes [17–19]. *K*-mer counting involves the use of short oligonucleotides to compare a sequence to either a reference genome or against genome of interest without needing an alignment [20]. The use of cgSTs or *k-*mers for differentiating *Campylobacter* genomes is based on the concept of genomic signatures and builds on the premise that infections originating from the same source are genetically more similar than those from different sources, facilitating the tracking of infections across various sources. Random forest models may use cgST data following the numerical encoding of alleles [21]. Encoding genes with the PCO-encoding method [22] incorporates information that quantifies the similarity between each pair of alleles and addresses issues related to missing alleles and new genotypes in observations for prediction. All models typically assume unidirectional flow from sources to sinks. However, a model to include intermediate nodes, which may act as both a source and a sink was developed to explore the role of water in the transmission of bacteria from livestock and water birds [23].

This study aims to assess the genomic diversity of *C. jejuni* in infants, humans and livestock in the Haramaya woreda, and to determine the attribution of infections in infants to livestock (chickens, cattle, goats and sheep) and other humans (siblings, mothers) on the basis of the genetic population structure of *C. jejuni* circulating in these reservoirs, using four different attribution models. This study was restricted to *C. jejuni* because the second dominant species in infants, *Candidatus* C. infans is not yet routinely culturable.

## Methods

### Isolation and sequencing of Campylobacter from human stool and livestock fecal samples

For the isolation of thermotolerant species by direct plating, one gram of fresh stool/animal feces was suspended in 9 ml buffered peptone water (pH 7; BD Difco). 100 µL of homogenized samples were spread on CHROMagar Campylobacter (CaC, DRG International, Springfield, New Jersey USA) using sterile glass beads and incubated for 48 h at 42 °C in microaerobic condition (85% nitrogen, 10% carbon dioxide, 5% oxygen) in anaerobic jars with GasPak EZ Campy Container System Sachets (ThermoFisher Scientific, Waltham, MA, USA). Similarly, for non-thermotolerant species, the same volumes of samples (100 µL) were plated on Columbia agar supplemented with 5% defibrinated sheep blood, Skirrow supplement (2 µL/mL), amphotericin B (5 µg/mL), cefoperazone (8 µg/mL) and *Campylobacter* growth supplement (ThermoFisher Scientific, Waltham, MA, USA). The plates were incubated at 37 °C for 48 h in microaerobic condition.

In parallel, samples were also enriched in Preston and Bolton broth with a proportion of 1 g feces in 9 ml of broth and incubator at 42 °C and 37 °C for 48 h, respectively, as described above. After incubation, 100 µL of Preston broth enriched samples were plated onto CaC and Bolton broth enriched samples on Colombia agar and plates were incubated at either 37 °C or 42 °C [24].

Due to global supply issues during the COVID-19 pandemic, we could not directly culture samples received before March 2022 and stored all samples to that date in 20% (w/v) glycerol at − 80 °C. This affected almost 85% (1857/2183) of the fecal/stool samples. Preliminary analysis indicated that up to 99% of the *Campylobacter* population in the feces could not be recovered on CaC within the first month of storage; therefore, samples were pre-enriched in Bolton broth before plating.

Typical *Campylobacter* colonies (up to 5 per plate) were sub-cultured onto a CaC plate and confirmed by genus-specific qPCR [25]. Potential thermotolerant and non-thermotolerant *Campylobacter* were characterized by streaking the confirmed pure isolate on to two fresh CaC plates and incubating at 37 °C and 42 °C in microaerophilic conditions for 48 h. The isolates growing at 42 °C and 37 °C were recorded as potentially thermotolerant while the isolates growing only at 37 °C were recorded as potentially non-thermotolerant. Despite our efforts to isolate non-thermotolerant *Campylobacter* species, we were only able to culture one non-thermotolerant isolate, later determined to be *C. hyointestinalis*. All isolates were stored in glycerol at − 80 °C.

For genomic DNA extraction, *Campylobacter* isolates from the freezer stock were grown on a CaC agar plate for approximately 36 h under microaerophilic condition at 42 °C. A loopful of growth was collected from the CaC plate, resuspended in 1 mL of Mueller Hinton broth (ThermoFisher Scientific, Waltham, MA, USA) and genomic DNA was extracted using Promega Wizard genomic DNA purification kit (Promega, Madison, WI, United States) following the manufacturer’s instructions. The concentration and quality of the DNA were determined using NanoDrop 2000 C Spectrophotometer (ThermoFisher Scientific, MA, USA). Purified DNA was shipped to eight GenomeTrakr Laboratories (FDA, USA) for sequencing.

All culturing and DNA extractions were performed in a dedicated laboratory at Haramaya University including physically separated spaces for sample reception, culturing, DNA extraction and PCR analysis as reported elsewhere [5]. The establishment of the laboratory, including back-up electricity and water supplies, was funded by Haramaya University and all equipment and supplies were sourced in the USA from the project budget and shipped under the responsibility of the University of Florida (UF). Laboratory staff were trained in person at The Ohio State University (OSU) and by in-person visits of experienced microbiologists from OSU and UF. During the COVID-19 pandemic, in-person visits were replaced by weekly conference calls and ad-hoc follow up calls as necessary.

Short-read genomic DNA libraries were prepared with the Illumina DNA prep kit, following the PulseNet Sequencing Protocol PNL35 [26]. Samples were sequenced using either paired-end 2 × 150 bp or 2 × 250 bp reads, which vary between sequencing laboratories (see Supplementary file MLST_profiles.xlsx for Biosample IDs). The paired-end reads were assessed for quality and contamination and trimmed using BBMerge (v.38.90) [27] and BBDuk (v.38.90) [28] with the following parameters: hammering distance 1, optimal *k*-mers 23, quality cutoff Q14 and minimum read length 30 bp, with end-trimming of a maximum 1of 0 bp. Species assignment was performed using KMC (version 3.0) [29] resulting in the identification of 380 *Campylobacter jejuni* isolates for analysis in this study.

### MLST assignment

Legacy MLST profiles (STs) for all *C. jejuni* isolates were determined using the *mlst* tool [30]. This utilizes the most recent update of the PubMLST database [31] (updated December 14, 2024), which incorporates the seven housekeeping loci scheme, as previously described [32]. We identified 11 novel STs and submitted these new schemes to PubMLST for the assignment of new STs. Core genome MLST (cgST) profiles were assigned using a 1,343-loci scheme (Cody et al. [19]), implemented through the *cgST* tool [33]. Missing alleles, which were unassigned, were identified using a custom R script and were assigned unique identifiers within the dataset. This approach ensured that all 1,343 loci were included in the analysis. The R script is publicly available at https://github.com/jmarshallnz/cgST. The sample set of 380 WGS samples included sequences collected from the same household and time point, representing different colonies obtained during subculturing in the pure isolation process. To create a unique representative dataset, we selected the isolates with the highest genomic coverage for each cgST type from each household and time point. This filtering resulted in a final WGS dataset comprising 287 isolates.

### MS tree construction and map

A minimum spanning tree (MST) was constructed using the GrapeTree plugin with the MSTreeV2 algorithm, which is designed to handle missing data more effectively than classical MST methods [34]. The process begins by calculating a directed minimal spanning arborescence using Edmonds’ algorithm from asymmetric distances, with tie-breaking based on allelic distances. Local branch recrafting was then performed to remove spurious branches.

### PERMANOVA

To explore the transmission of *C. jejuni* at different levels within the sampling hierarchy we performed a nested, permutational multivariate analysis of variance (PERMANOVA) [35] to estimate the proportion of variance in infant cgST profiles attributable to each level, namely sample (i.e., multiple isolates from the same infant sample), infant (i.e., multiple isolates from the same infant at different time points), ganda (village) and kebele (the smallest administrative unit in Ethiopia, a set of gandas). PERMANOVA models were constructed using a customised R script (https://github.com/jmarshallnz/permanova). Pairwise genetic distances were calculated from the cgST profiles to create a distance matrix with values in the matrix corresponding to the Gower distance calculated using the vegdist() function in the R package *vegan* [36]. Multiple two-level nested models were considered: *infant within ganda*, *ganda within kebele*, *infant within kebele* and *infant time point within infant*. It was not possible to fit models considering higher-level nested structures, so only two-level nested models are presented. Univariate PERMANOVA models were performed for each factor with *p-value*s obtained using 100,000 unrestricted permutations of raw data.

### Diversity and persistence of C. jejuni infections

The isolate set included up to four isolates from the same infant sample, while 25 sets of repeat samples from the same infant at different timepoints (approximately monthly intervals) were also available. We quantified the diversity of *C. jejuni* isolates in these samples by counting the number of isolates by ST/cgST using a bespoke coding system in Excel (Supplementary file MLST_profiles.xlsx). Similarly, the occurrence of the same or different STs/cgSTs in sequential samples from the same infant was quantified and summarized in relation to the time interval between two samples using pivot tables in Excel.

### Attribution

While livestock are commonly recognized as the main sources of human infections, humans other than infants are exposed to the same contaminated environment as the infants are, and they can be considered either as independent receiving hosts or as receiving, amplifying hosts that transmit infection to infants. Exclusive human-to-human transmission cycles cannot be excluded a priori. We therefore fitted models with only livestock sources as well as models with other humans as additional sources of infections in infants.

A summary of all fitted models is provided in Table 1. All analyses were performed in the statistical language R version 4.3.0 or later [37] or Excel (Microsoft Corporation, Redmond, WA), using dedicated R packages or other software as indicated in the text.

**Table 1:** Overview of source attribution models

Source attribution model	Input data	Summary data	Attribution pathways	Model fitting procedure
Asymmetric Island	Assembled contigs	cgST^*^ profiles	Direct	MCMC^&^
*k*-mer	Raw reads	9-mers	Direct	Random Forest with feature reduction
*PCO*	Assembled contigs	cgST profiles + allele sequences	Direct	Random Forest without feature reduction
Source-sink	Assembled contigs	cgST profiles	Direct and indirect	MCMC

*core genome Sequence Typing

^&^Markov Chain Monte Carlo

#### Asymmetric Island model

The asymmetric-island source attribution model [14] was applied to the cgST data for 287 isolates using the *islandR* package (https://github.com/jmarshallnz/islandR). This method assigns sources to isolates by modeling both recombination and mutation processes as distinct events. In our analysis, the recombination and mutation probabilities were assumed to be constant across all sources, resulting in pooled estimates for both processes. The model incorporates genetic differences between isolates to estimate the most likely source, accounting for multiple potential sources. From the model estimates, the mean and 95% confidence intervals of the posterior distribution were calculated to determine the attribution percentages for each source. This approach is particularly useful for including recombination and mutation in source attribution, enhancing the accuracy of source estimation based on cgST profiles.

#### PCO model

The PCO source attribution model is a random forest model that uses a principal coordinates approach to overcome the problem of missing levels in the data used for prediction. This method uses a target-agnostic approach to encode cgST predictor variables by using both the cgST allele profiles and Hamming distances between allele sequences to determine the similarity between pairs of isolates [22]. Analyses were carried out using the *ranger* package [38] and the PCO-encoding method (https://github.com/smithhelen/LostInTheForest). Estimates of uncertainty are calculated using a probability forest with the same set of parameters as the original random forest. For each tree in the forest, the probability of each human isolate being attributed to each source is calculated. These probabilities are then averaged over the set of infant isolates, giving an average probability of attribution to each source for each tree. The 2.5% and 97.5% quantiles are then determined from this set of mean probabilities to give a 95% uncertainty interval.

#### Source/sink model

The role of humans as intermediate hosts for zoonotic infections was further explored using a model in which they can act both as sources and as a sink. The IslandR model was reparametrized to consider the mothers and siblings as both receiving infection from the animal reservoirs and being a source of infection for infants. The source/sink model was based on an approach developed to examine the contribution of water as both a source and a sink for human campylobacteriosis in New Zealand [23]. In essence, we assume that mother and sibling isolates arise from a mix of the animal reservoirs, whereas infant isolates arise from a mix of both the animal reservoirs and mothers and siblings. This model is fit using the attribution_intermediate() function in the *islandR* package (https://github.com/jmarshallnz/islandR).

#### k-mer model

The tool KMC (version 3.0) [29] was used to extract *k-*mers with length of k = 9 for each of the samples using the short-read sequences. All *k*-mer frequencies were then combined into one matrix using an in-house Python script. A recently developed [21] source attribution model was applied to the two datasets using the *k*-mers to predict the sources of human campylobacteriosis cases. Feature reduction was carried out on the matrix to reduce the number of *k*-mers in the final model using the *caret* package (version 6.0–94) [39] and the *Boruta* package (version 8.0.0) [40]. The near-zero-variance method was used to reduce the number of 9-mers. The Boruta algorithm was then applied to select important attributes in the matrix using a random forest classifier. To account for the uneven distribution of sources in the samples, all sources were upsampled to the highest number of samples available within a source, so that all sources had the same number of samples. Two machine-learning algorithms previously applied successfully in sequencing studies were evaluated [41–44]. For the evaluation, the data containing *k*-mers from sources were split into test- and training data sets. The training data were then used to randomly generate smaller sets of test and training data once again to determine which of the two selected machine-learning algorithms fit the data best. Each smaller test- and training data set was split 70% and 30%, respectively, and the test-data were used to evaluate the performance of the model using seven-fold cross-validation. After 10 iterations, the accuracy of each algorithm was assessed, and the algorithm with the highest accuracy was selected for model construction.

The model with the highest accuracy was constructed again following the same steps as for the model selection, described previously, and the performance of the model was evaluated based on the accuracy of the cross-validation step, the kappa value and the confusion matrix, which determines the model’s ability to predict the sources of the samples in the source-data. The sensitivity and specificity were also reported. Finally, the model was applied to isolates from infants. This was done by estimating the probability of each human case being attributed to each of the sources included in the model. Tree-level predictions were pooled together to calculate the mean probability for each source, with 2.5th and 97.5th percentiles providing uncertainty intervals for each case. To estimate the uncertainty in the overall mean attribution probabilities, we performed 1,000 bootstrap resampling iterations. In each iteration, case isolates were sampled with replacement, and mean attribution probabilities were recalculated. The 2.5th and 97.5th percentiles of the bootstrap distributions provided 95% uncertainty intervals for the mean probabilities.

## Results

We included WGS data from 380 isolates for attribution from different sources analysis by *k*-mers (Table 1) and of these, assigned cgST profiles to 287 isolates. The majority of human samples other than those from infants were collected from siblings and the majority of livestock samples were collected from chickens with fewer samples from ruminant species. Owing to the small number of isolates from several sources, we ran attribution models of infections in infants with two merged source groups: other humans (mothers and siblings) and small ruminants (sheep and goats). Cattle are often recognized as a major reservoir for *Campylobacter* transmission to humans, particularly *C. jejuni*. By keeping cattle separate, their unique role as a distinct source is emphasized. Additionally, other studies and risk assessments use the "small ruminant" classification for sheep and goats because of their shared characteristics. This convention supports comparability and consistency across studies.

### Genomic diversity of *C. jejuni* from different sources

**Fig. 1:**
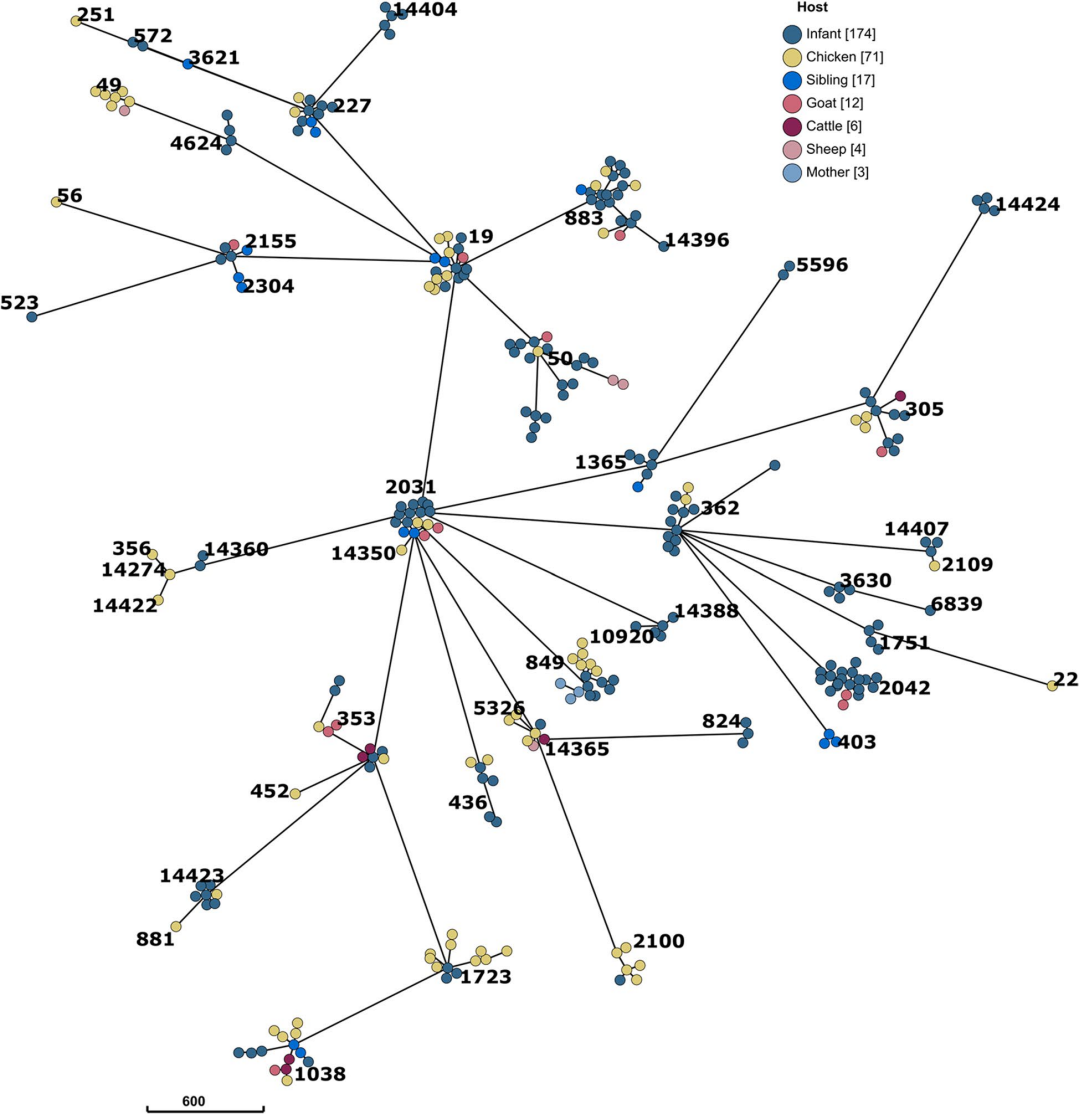
Minimum spanning tree illustrating the distribution of *Campylobacter jejuni* core genome ST types among human and livestock isolates from Ethiopia. Nodes are color-coded according to host type (human or livestock), and each node represents a unique isolate. Solid lines between nodes indicate phylogenetic relatedness, and scale bar represents a 600-loci distance. Clusters of nodes sharing identical ST types are labeled with the corresponding ST sequence type in black text

The set of 287 isolates included 48 STs. The population structure based on cgST is presented as a minimum spanning tree (MSTree) in Fig. 1. The tree is fully structured according to seven-gene STs and isolates within the same ST are highly related, differing by fewer than five alleles. There were 11 newly assigned STs (37 isolates; see Table S1). Among these, one type was common to infants, chickens, cattle and sheep; one was common to infants and chickens; six were unique to infants; and three were unique to chickens (Table S2). Twelve STs were represented by more than 10 isolates, which together accounted for almost two-thirds (185/287) of all the isolates. Isolates from six of these STs were shared among infants, livestock and other humans, whereas six were shared only between infants and livestock. Detailed data on these isolates are available in the Supplementary file MLST_profiles.xlsx.

### Diversity and persistence of C. jejuni infections

We obtained multiple isolates of *C. jejuni* from 81 samples. Of these, one sample yielded four isolates, 34 (28 + 5 + 1) samples yielded three isolates, and 46 (*39 + 7) samples yielded two isolates. All isolates from the same sample were of the same seven-gene ST in 84% (68/71) of the samples (Table 2 and supplementary file MLST_profiles.xlsx), thus not providing evidence of diversity of STs within the host. Two or three different STs were found in 9 samples, suggesting sequence type diversity within the host. The cgST profiles of all the isolates from the same sample within the same ST were fewer than five alleles different.

**Table 2:** *C. jejuni* isolates included in the k-mer and cgST attribution models

Source group	Source	k-mer data set	Grouped k-mer data set	cgST data set	Grouped cgST data set
Infants		229	229	174	174
Other Humans	Mothers	3	29	3	20
	Siblings	26		17	
	Chickens	86	86	71	71
	Cattle	13	13	6	6
Small Ruminants	Sheep	2	24	4	16
	Goats	22		12	
Total		380	380	287	287

Persistence of *C. jejuni* infections (i.e., isolation of the same ST from two sequential samples) was observed in 8% (2/25) of sample pairs from the same infant (supplementary file MLST_profiles.xlsx). These pairs were taken approximately 1 or 2 months apart. Different STs were observed in 84% of the sample pairs, the majority of these STs were also 1–2 months apart, but five pairs had 3-months intervals and 1 pair had a 4-month interval. Eight percent (2/25) of sample pairs (1- or 2-month intervals) provided inconclusive evidence with the same STs being isolated in both samples, accompanied by one or more different STs.

### Spatial clustering

**Fig. 2:**
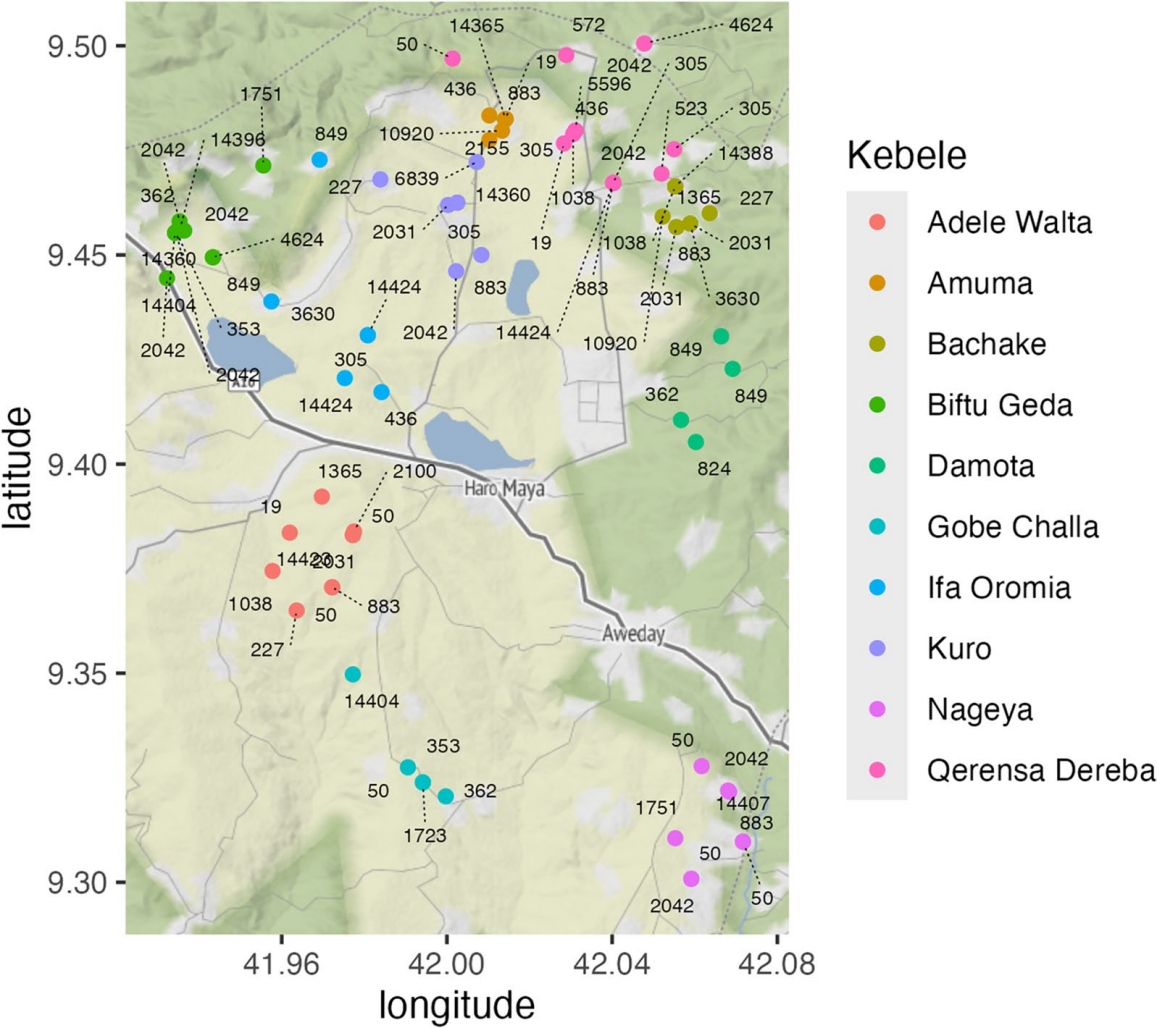
Spatial distribution of STs from infants in Haramaya woreda, Ethiopia. Solid blue lines indicate kebele boundaries, dots indicate geographic position of STs. Shading indicates vegetation density. Haramaya University is in the center of the map, just north of Haro Maya city. Map constructed with *ggmap*, based on OpenStreetMaps

Figure 2 shows the spatial distribution of STs from infants. Common STs, such as ST50, ST883 and ST2042 were found in multiple gandas (villages) and kebeles (the smallest administrative unit in Ethiopia). We analyzed the distribution of cgST types at different nested levels of spatial and temporal sampling using PERMANOVA, estimating the contribution of the variation in cgST alleles attributable to kebeles, gandas, infants at different time points and infant samples. We tested for significant clustering at each level, using multiple two-level nested models (Table S3).

Considering *infants within kebeles*, some 14% of the total variation in cgST profiles was between kebeles. Most of the total variation was between *infants within kebeles* (54%), and 33% of the variation was *within infants*. There was marginally significant clustering at the kebele level (p = 0.03) and highly significant clustering at the infant level (p < 0.001).

Considering *gandas within kebeles*, some 14% of the total variation in cgST profiles was between kebeles. Most of the total variation was between *gandas within kebeles* (47%), and 40% of the variation was *within gandas*. There was a tendency toward clustering at the kebele level (p = 0.053) and highly significant clustering at the ganda level (p < 0.001).

Considering *infants within gandas*, some 58% of the total variation in cgST profiles was between gandas. The total variation between *infants within gandas* was 9%, and 33% of the variation was *within infants*. There was no significant clustering at the ganda level (p = 0.29) but there was highly significant clustering at the infant level (p < 0.001).

Considering *samples within infants*, some 68% of the total variation in cgST profiles was between infants. The total variation between *samples within infants* was 22%, and 10% of the variation was *within infants*. There was significant clustering at the infant level (p < 0.01) and highly significant clustering at the sample level (p < 0.001).

The highly significant clustering at the sample level confirms observations in Table 3 of highly related isolates from one sample. We, therefore, repeated the analysis, using a reduced dataset including only unique STs per sample (97 isolates). The results of the partitioning of variance at all levels of clustering were very similar to those of the full dataset. However, the significance of clustering at the *infants within gandas* changed markedly to no significant clustering at both the ganda and infant levels.

**Table 3:** Multi-Locus Sequence Type diversity in human and livestock samples

Isolate pattern^*^	Infant	Sibling	Mother	Cattle	Goat	Chicken	Sheep	Total
A1234^&^	1	0	0	0	0	0	0	1
A123^&^	25	1	1	0	0	1	0	28
A12^&^	22	5	0	2	2	7	1	39
A12B^#^	5	0	0	0	0	0	0	5
AB^#^	3	0	0	0	0	4	0	7
ABC^#^	1	0	0	0	0	0	0	1
*Evidence of sequence type diversity*								
No^&^	48	6	1	2	2	8	1	68
Yes^#^	9	0	0	0	0	4	0	13
Total	57	6	1	2	2	12		81

*Letters indicate different seven-gene STs, and numbers indicate different cgSTs among isolates from one ST. The number of isolates differs between samples and is represented by the number of different letter/number combinations. For example, A1234 indicates four isolates from one sample with the same ST, but all different cgSTs, whereas A12B indicates three isolates from one sample of which two with the same ST but different cgSTs, and one isolate with a different ST (and consequently also a different cgST). Subscripts indicate patterns with^#^ or without^&^ evidence of sequence type diversity

### Attribution

**Fig. 3:**
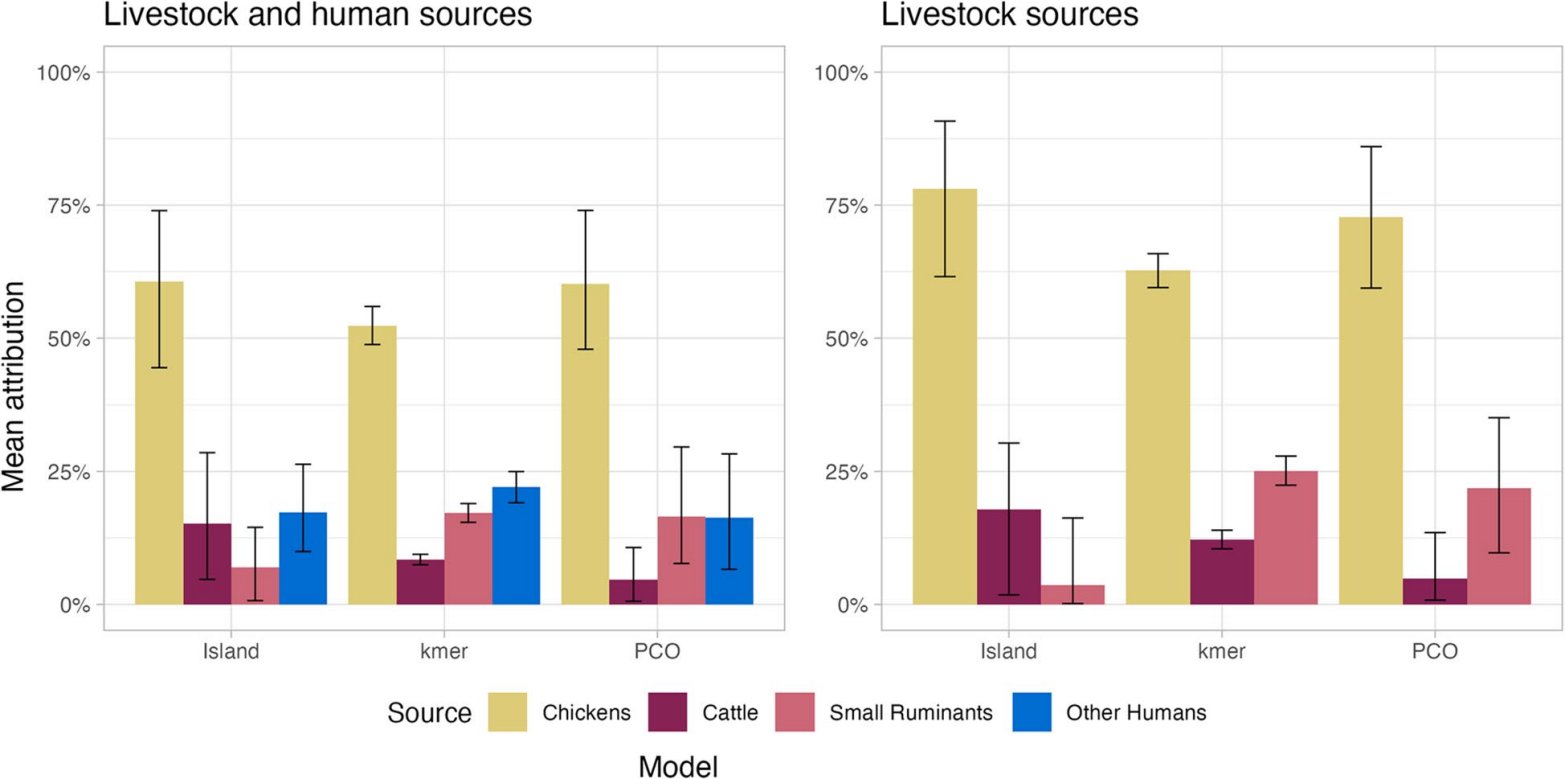
Attribution estimates for *C. jejuni* infections of infants to livestock and human sources using Asymmetric Island, PCO and *k*-mer models

The attribution results for the Asymmetric Island, PCO and *k*-mer models are summarized in Fig. 3 and Table 4.

**Table 4:** Attribution estimates for *C. jejuni* infections of infants to livestock and human sources using Asymmetric Island, PCO and *k*-mer models

Models and source categories	Attribution percentage			
	Chickens	Cattle	Small ruminants (goats and sheep)	Other humans (mothers and siblings)
Asymmetric Island model				
Livestock and humans	60.6^*^ (44.4–74.6)	15.2 (4.7–28.5)	6.9 (0.1–14.5)	17.3 (10.0–26.4)
Livestock	78.1 (61.6–90.1)	17.8 (0.1–30.3)	3.6 (0.0–16.3)	–
PCO model				
Livestock and humans	60.2 (47.9–74.0)	4.6 (0.1–10.7)	16.5 (0.1–29.6)	16.3 (0.1–28.3)
Livestock	72.7 (59.4–86.0)	4.9 (0.1–13.5)	21.9 (0.1–35.1)	–
*k*-mer model				
Livestock and humans	52.3 (48.8–55.9)	8.4 (7.5–9.4)	17.2 (15.4–19.0)	22.0 (19.1–25.0)
Livestock	62.8 (59.5–65.9)	12.1 (10.4–14.0)	25.0 (22.4–27.9)	–

*Mean (95% uncertainty interval)

The PCO random forest model was trained on the set of source isolates using all 1343 cgST genes (as nominal predictors). The genes were encoded using the PCO-encoding method together with a dissimilarity matrix of Hamming distances of the nucleotide sequencing information between each pair of alleles for each gene. Any new alleles in the set of human isolates for prediction were encoded using the method of principal coordinates based on pairwise Hamming distance from the new alleles to the alleles in the set of source isolates. The original sources of the infant isolates were then predicted.

The *k*-mer models were built on 131,073 attributes (or 9-mers), which were then reduced further by the near-zero variance method which removed few attributes from the model, depending on which dataset was modeled. The Boruta algorithm further reduced the matrix by selecting only those that are confirmed to provide enough important information to be included in the model.

For the dataset including both humans and animals as sources, the near-zero variance method identified two attributes with low variance, which were removed from the data set. The Boruta algorithm further reduced the dataset to include 69 important attributes used for further modeling. For the dataset including only animal sources, the near zero variance method removed one attribute with low variance, whereas the Boruta algorithm further reduced the number of attributes to 23 used for further modeling. For both datasets, the performances of the random forest and the logit-boost algorithms were compared (Table S4). The average accuracies obtained from taking the average across ten iterations showed that the random forest and the logit boost algorithms performed very similarly in terms of accuracy for both data sets. The logit boost performed marginally better but could not provide uncertainty intervals comparable to the other approaches. Consequently, we decided to use the random forests algorithm.

The final models predicted probabilities for each of the *C. jejuni* infections of infants to originate from each of the sources (Table 4). The results were similar for all models with most cases being attributed to chickens. The asymmetric Island model attributed most infections among ruminant sources to cattle, whereas the PCO and *k*-mer models (both machine-learning models) considered small ruminants more likely. The percentage of cases attributed to chickens was lower for the *k*-mer model than for the other two models, both of which use cgST for genomic characterization. Although both the *k*-mer and the PCO models are random forest models, the *k*-mer method uses feature reduction to substantially decrease the number of variables in the model. The smaller pool of predictors means there is less variation in each tree of the forest, which potentially explains the smaller uncertainty intervals. When other humans are included as putative sources, the models estimate that some 16–22% of all infant isolates originate from other humans, reducing mainly the estimate for chickens.

**Fig. 4:**
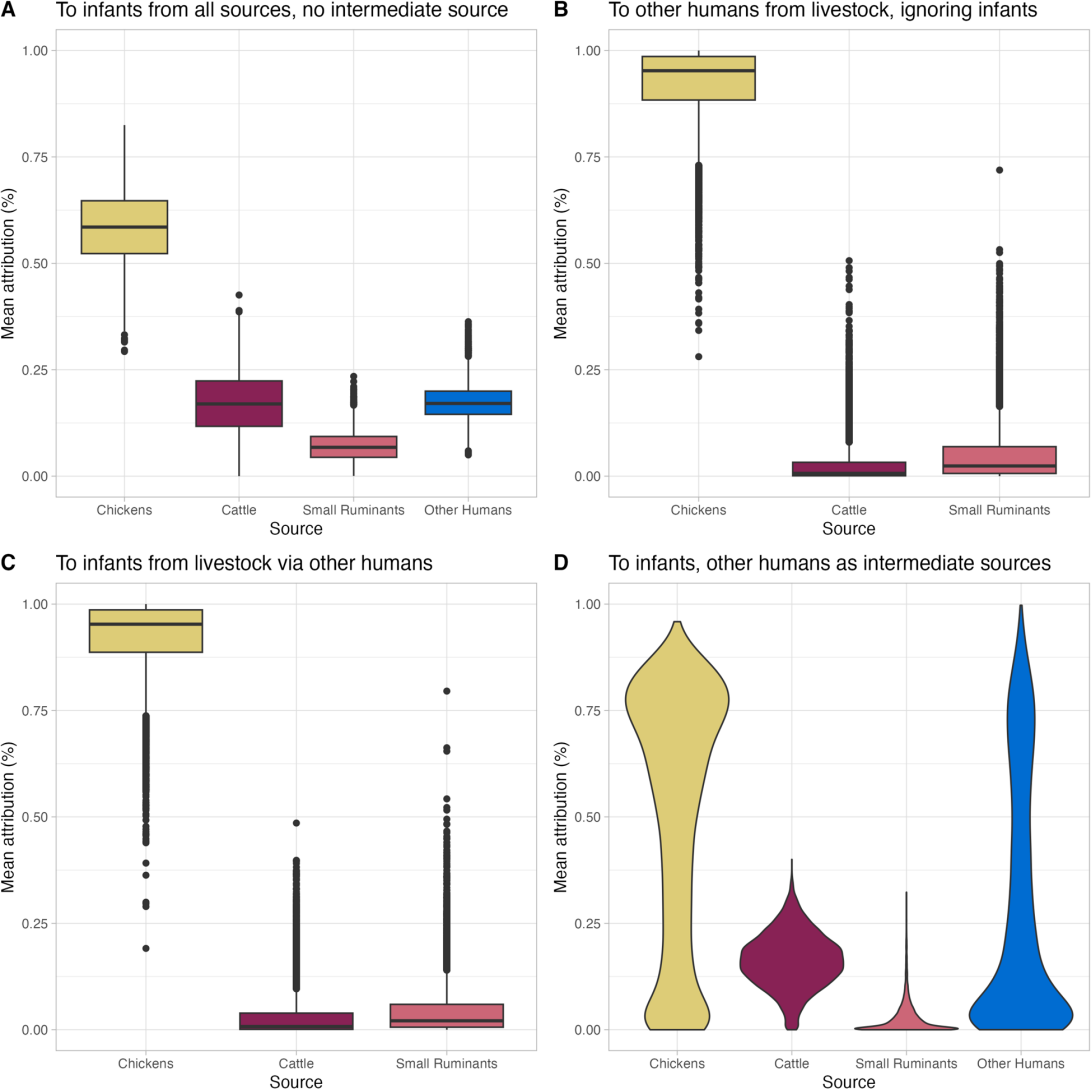
Attribution estimates for *C. jejuni* infections of infants to livestock and human sources using the source-sink model

The source/sink model was run for different source combinations (Fig. 4 and Table 5). When considering infections of infants from all putative sources independently, the results were similar to those of the Island model, as expected. When considering infections in mothers and siblings from livestock sources, the attribution was almost exclusively to chickens with very low percentages attributed to cattle or small ruminants. Likewise, if mothers and siblings were considered as intermediate sources between livestock and infants, chickens were identified as the main source which then gets passed on to infants. This results in a lack of identifiability for the attribution to infants when aiming to separate the direct chicken route from the indirect chicken route through mothers and siblings (Fig. 4d).

**Table 5:** Attribution estimates for *C. jejuni* infections of infants to livestock and human sources using the source-sink model

Models and source categories	Attribution percentage			
	Chickens	Cattle	Small Ruminants (goats and sheep)	Other Humans (mothers and siblings)
To infants from all sources, no intermediate source				
Livestock and humans	58.4^*^ (42.6–73.5)	17.2 (3.9–31.1)	7.0 (1.1–14.4)	17.4 (10.3–26.3)
To mothers and siblings from livestock, ignoring infants				
Livestock	92.2 (67.4–99.9)	2.9 (0.0–18.5)	5.1 (0.0–25.1)	–
To infants from livestock via mother and siblings				
Livestock	92.2 (69.3–99.9)	3.3 (0.0–20.1)	4.5 (0.0–21.6)	–
To infants from all sources, mothers and siblings as intermediate sources				
Livestock and humans	50.9 (0.3–88.0)	16.2 (2.6–29.0)	2.8 (0.0–14.7)	30.0 (0.0–86.8)

*Mean (95% uncertainty interval)

## Discussion

We used whole-genome-sequencing data to analyze the genomic diversity of *C. jejuni*, and to attribute infections to putative livestock and human sources. Among the 287 isolates, 48 STs were identified, among which 11 were previously unreported types. In a meta-analysis of global genotypes of *C. jejuni*, Poorrashidi et al. [45] reported that the six most common STs globally are ST21, ST45, ST50, ST48, and ST257. Among these STs, only ST50 was found in our study as the most frequent ST. Many of the isolates recovered in this study do not belong to the most common STs reported in high-resource settings, and many have never been reported before. This is likely to be due to sampling bias and limited application of WGS in low-resource settings [46]. Of the 87,542 genomes of *Campylobacter* in the PubMLST database, only 386 are from Africa (https://pubmlst.org/bigsdb?db=pubmlst_campylobacter_isolates&page=query&genomes=1, accessed December 12, 2024).

*C. jejuni* ST50 isolates have been frequently reported globally from environmental, food and clinical sources. Poultry isolates from Oceania, Europe and North America tended to cluster on the basis of the continent where the sample was collected [47]. No isolates from Africa were included in this study. Other frequent STs in our study are ST883, ST19, ST20242 and ST2031. The two former STs were found in infants and goats in our study, have previously been found in humans (children and mothers) in Ethiopia [48] and have been associated with a raw cow’s milk outbreak in Finland (ST883) [49] and Denmark (ST19) [50], respectively.

Several studies have detected a high prevalence of *C. jejuni* in human, livestock and food samples from Ethiopia, but only two studies have included the genomic characterization of isolates. Among 14 isolates from dairy in the Ethiopian regions of Amhara, Oromia, and the Southern Nations, Nationalities, and Peoples (SNNPR) between 2020 and 2021, two STs (ST51 and ST2084) were detected [51]; neither of these types was identified in our study. Another study from the Harar town and Kersa district identified 8 distinct STs for 19 Campylobacter strains isolated from children, caretakers and potential exposure sources [48]. The most abundant STs were ST353, ST19 and ST1365, all of which were also found in this study. French et al. [52] studied the genomic structure of poultry isolates from Tanzania and human isolates from Kenya, and reported that ST353, ST8043, ST2122 and ST1932 were the dominant STs (in this order). Of these, only ST353 was identified in the current study as one of the twelve most frequently occurring types. In both studies, these types were isolated from both humans and livestock. These authors also tabulated STs as identified in other studies from Africa. Among the twelve most common STs identified in our study, ST362 was identified frequently in South Africa, whereas some other STs occurred sporadically in the database. While the current results suggest little overlap between types in geographically adjacent regions, the genomic diversity of *C. jejuni* and other foodborne pathogens in Africa is largely uncharacterized. More generally, WGS-based surveillance is poorly developed in low-resource countries [46].

Our isolate collection included several samples from which multiple isolates were obtained, offering a unique opportunity to assess genomic diversity in single hosts. Among stool samples from infants, we detected up to four isolates with the same seven-gene ST and only a few samples with more than one ST. Within one ST, cgSTs had fewer than five alleles difference. Bloomfield et al. [53] studied sixteen isolates from an immunodeficient patient who was colonized by *C. jejuni* for 10 years after an episode of diarrhea. All isolates shared a common ancestor, coinciding with the onset of symptoms for the patient and evidence was found for genetic bottlenecks due to antimicrobial treatment. Djeghout et al. [54] sequenced ninety-two *C. jejuni* isolates from four different patients with gastroenteritis and used Single Nucleotide Polymorphisms (SNP) to assess phylogenetic relationships. Three patients yielded a single seven-gene ST, whereas one patient yielded two different STs. Isolates from one patient were genetically diverse, even within one ST (12–43 core non-recombinant SNPs and 0–20 frame-shifts). These authors concluded that this diverse population was unlikely to have evolved from a single isolate at the time point of initial patient infection and that patients were likely infected with a heterogeneous *C. jejuni* population. However, even upon exposure to a heterogeneous population, multiple barriers in the host create a strong evolutionary bottleneck, resulting in the selection of one single variant causing colonization except when the host is exposed to high doses [55]. We therefore suggest that in our population of young infants, infections are caused mainly by a single transmission event, followed by microevolution within the host. Further phylogenetic analysis of our isolates including Average Nucleotide Identity and Single Nucleotide Polymorphisms may provide more detailed information but was beyond the scope of this study.

A reanalysis of data from the MAL-ED birth cohort study suggested that persistent infections with *Campylobacter* were associated with poorer 9-month linear growth. Persistent infections were defined as three or more consecutive *Campylobacter* positive monthly stools by qPCR [56]. However, in the main text the authors state that “persistent Campylobacter infections cannot be differentiated from recurrent reinfections with epidemiologic data alone” and suggest the term persistent carriage would better describe the qPCR results. Because of the longitudinal nature of our study, we were able to evaluate temporal patterns of genotype diversity in infants. Repeat isolates of the same ST at two different time points (1 or 2 months apart) were observed but in most cases, we observed different STs. This suggests a dominant pattern of clearance of one type and new infection by other types within a time interval of one to 4 months, which is consistent with findings from a Markov Chain model applied to the same MAL-ED dataset [57]. These findings are further supported by PERMANOVA, indicating a high level of clustering of isolates from the same sample, but no significant clustering of isolates from the same infant at different time points. The clustering at the kebele level suggests that there is localized, within kebele transmission which is independent of the clustering observed within samples and between samples from the same individual over time.

We used two different methods (cgST and *k*-merization) to characterize the genomic diversity of our isolates and two types of models (population genetics and machine learning) to attribute infections in infants to putative sources. Chickens were the main source of infection with model uncertainty in the proportion of infections attributed to cattle or small ruminants. When other humans are included as possible sources, attribution to livestock decreases but chickens remain the main source. Neither the Asymmetric Island method using cgST nor the random forest methods using cgST or *k*-mers can determine directionality. The assumption is that there is unidirectional flow from any of the included sources to the receiving host (sink). The “source-sink” model relaxes this assumption in the sense that some compartments are considered both as sources and as sinks. This model was originally applied to evaluate the role of water as a sink for contamination by water birds and as a source for human infections [23]. We applied this model to study the role of other humans as sinks from infections from livestock and as sources for infections in infants. The model suggested that other humans are largely infected by chickens, and that these infections may be transmitted to infants. We cannot conclude whether other humans act as mechanical vectors or if they are amplifying hosts.

Our source attribution results may be biased towards chickens because of the substantially greater number of isolates than those from other livestock and human sources. For example, isolates from goats and siblings frequently occurred among the top twelve STs but less frequently among rare STs. Nevertheless, the use of high-resolution genome sequencing data, and the similar findings from multiple models based on different underlying assumptions, suggest that any bias attributed to unbalanced sampling is unlikely to affect the conclusion that chickens are the most important animal reservoir for infant infections in this study. Nevertheless, as a more important role of ruminants cannot be ruled out, control strategies aimed at eliminating transmission from the chicken reservoir only may not be effective. Additionally, the degree of transmission between chickens and ruminants is unknown but is likely to occur, particularly in areas where the animals are not confined to barns or other housing facilities.

Notably, most of the isolates were from older infants, because of increasing prevalence with age. Furthermore, this study was undertaken during the global COVID-19 pandemic and early samples could not be cultured because of global supply chain issues and samples were preserved by freezing. Even though we stored the samples in glycerol, recovery was strongly affected. Additionally, species-specific qPCR results indicated that *Candidatus C. infans* is more prevalent in infants than *C. jejuni* is (70% at 1 year of age). This species has mainly been detected in other humans, with low levels of detection in livestock [8], suggesting this species is anthroponotic in nature with occurrence in livestock as a reverse zoonosis, or even merely passing through the animal gut from a highly contaminated environment, where open defecation is common [6].

The transmission pathways of *C. jejuni* in our study area are highly complex and interdependent. The EXCAM study enrolled 79 participating households from the participants in this study and in the same time frame employed behavioral observations, microbiological analysis and mathematical modeling to create an agent-based exposure model framework to quantify the exposure to generic *Escherichia coli* through different pathways in the first and second half years of life of the infants. The major sources of exposure to *E. coli* were food and breastfeeding in the first half year of life and food and soil in the second half year of life. Caretakers’ hands are the main sources of contamination of both food and breast surfaces [58].

## Conclusions

Many of the *C. jejuni* isolates identified in this study do not belong to the most common STs reported in high-resource settings. Among the six most common global STs, only one was found in our study area. Isolates from the same infant sample were highly related, isolates from consecutive infant samples usually had different STs, suggesting rapid clearance and new infection. The transmission pathways of *C. jejuni* in our study area are highly complex and interdependent. While chickens are the most important reservoir of *C. jejuni* in infants, ruminant reservoirs also contribute to the infections. Model predictions differed in terms of the relative importance of cattle versus small ruminants as additional sources. Infections from chickens are transmitted with or without other humans (mothers, siblings) as intermediate sources and the role of human–human transmission in infancy needs to be further elucidated. To reduce the colonization of infants with *Campylobacter* and ultimately mitigate stunting in low resource settings, we recommend to initially focus on protecting proximate sources. This includes ensuring the cleanliness of caretakers’ hands food and indoor soil Achieving these goals requires a tight integration of the currently siloed domains of nutrition, food safety and water, sanitation and hygiene.

## Supplementary Information

Additional file 1

Additional file 2

## Data Availability

All whole-genome sequences supporting the conclusions of this article are available under Bioproject PRJNA1015272 at https://www.ncbi.nlm.nih.gov/bioproject/?term=PRJNA1015272. Biosample IDs for selected *Campylobacter jejuni* isolates, post-processing, are provided in the supplementary file MLST_profiles.xlsx. Code on GitHub is indicated in the text.

## Abbreviations

CaC: Chromagar Campylobacter
cgST: Core genome Sequence Typing
cgST: Core genome Sequence Type
*k*-mer: Substring of length k contained within a DNA sequence
PCO: Ordinal encoding of predictor variables using Principal Components
MST: Minimum Spanning Tree
PERMANOVA: Nested, permutational multivariate analysis of variance
PubMLST: Public databases for molecular typing and microbial genome diversity
qPCR: Quantitative (real-time) Polymerase Chain Reaction
SNP: Single Nucleotide Polymorphism
ST: Legacy (seven-gene) Multi Locus Sequence Type

## References

[1] Havelaar AH, et al. World Health Organization global estimates and regional comparisons of the burden of foodborne disease in 2010. PLoS Med. 2015;12: e1001923. 26633896 10.1371/journal.pmed.1001923 PMC4668832

[2] Hald T, et al. World Health Organization estimates of the relative contributions of food to the burden of disease due to selected foodborne hazards: a structured expert elicitation. PLoS ONE. 2016;11: e0145839. 26784029 10.1371/journal.pone.0145839 PMC4718673

[3] Rogawski ET, et al. Use of quantitative molecular diagnostic methods to investigate the effect of enteropathogen infections on linear growth in children in low-resource settings: longitudinal analysis of results from the MAL-ED cohort study. Lancet Glob Health. 2018;6:e1319–28. 30287125 10.1016/S2214-109X(18)30351-6 PMC6227248

[4] Hendrickson SM, et al. *Campylobacter* vaccination reduces diarrheal disease and infant growth stunting among rhesus macaques. Nat Commun. 2023;14:3806. 37365162 10.1038/s41467-023-39433-1 PMC10293212

[5] Deblais L, et al. Prevalence and load of the *Campylobacter* genus in infants and associated household contacts in rural eastern ethiopia: a longitudinal study from the campylobacter genomics and environmental enteric dysfunction (CAGED) project. Appl Environ Microbiol. 2023;89:e00424-e523. 37310259 10.1128/aem.00424-23 PMC10370295

[6] Chen D, et al. *Campylobacter* colonization and undernutrition in infants in rural Eastern Ethiopia: a longitudinal community-based birth cohort study. medRxiv. 2024. 10.1101/2024.05.21.24307707. 39839388 10.3389/fpubh.2024.1467462 PMC11747651

[7] Li X. et al. (2024) Geospatial analysis of multilevel socio-environmental factors impacting the *Campylobacter* burden among infants in rural eastern Ethiopia: a one health perspective. 2024.07.03.24309853 Preprint at 10.1101/2024.07.03.24309853. 10.4269/ajtmh.24-0401 PMC11884283 39742520

[8] Ojeda A. et al. (2025) Determinants of *Campylobacter* species diversity in infants and association with family members, livestock, and household environments in rural Eastern Ethiopia. Submitted to Gut Pathogens. 10.1186/s13099-025-00725-0 PMC12228300 40618155

[9] Mekuria Z. et al. (2025) Host clustering of *Campylobacter* species and other enteric pathogens in a longitudinal cohort of infants, family members and livestock in rural eastern Ethiopia. Submitted for publication. 10.1186/s40168-025-02203-w PMC12581456 41185061

[10] Cody AJ, Maiden MC, Strachan NJ, McCarthy ND. A systematic review of source attribution of human campylobacteriosis using multilocus sequence typing. Eurosurveillance. 2019;24:1800696. 31662159 10.2807/1560-7917.ES.2019.24.43.1800696 PMC6820127

[11] Van Pelt W. et al. Origin, extent and costs of human salmonellosis. Part 1 Origin of human salmonellosis with respect to pig, cattle, chicken, eggs and other sources (in Dutch). Infectieziekten Bulletin 10.

[12] Hald T, Vose D, Wegener HC, Koupeev T. A Bayesian approach to quantify the contribution of animal-food sources to human salmonellosis. Risk Anal. 2004;24:255–69. 15028016 10.1111/j.0272-4332.2004.00427.x

[13] Mullner P, et al. Source attribution of food-borne zoonoses in new Zealand: a modified hald model. Risk Anal. 2009;29:970–84. 19486473 10.1111/j.1539-6924.2009.01224.x

[14] Wilson DJ, et al. Tracing the source of campylobacteriosis. PLoS Genet. 2008;4: e1000203. 18818764 10.1371/journal.pgen.1000203 PMC2538567

[15] Pritchard JK, Stephens M, Donnelly P. Inference of population structure using multilocus genotype data. Genetics. 2000;155:945–59. 10835412 10.1093/genetics/155.2.945 PMC1461096

[16] Arning N, Sheppard SK, Bayliss S, Clifton DA, Wilson DJ. Machine learning to predict the source of campylobacteriosis using whole genome data. PLoS Genet. 2021;17: e1009436. 34662334 10.1371/journal.pgen.1009436 PMC8553134

[17] Bernard G, Chan CX, Ragan MA. Alignment-free microbial phylogenomics under scenarios of sequence divergence, genome rearrangement and lateral genetic transfer. Sci Rep. 2016;6:28970. 27363362 10.1038/srep28970 PMC4929450

[18] Panyukov VV, Kiselev SS, Ozoline ON. Unique k-mers as strain-specific barcodes for phylogenetic analysis and natural microbiome profiling. Int J Mol Sci. 2020;21:944. 32023871 10.3390/ijms21030944 PMC7037511

[19] Cody AJ, Bray JE, Jolley KA, McCarthy ND, Maiden MCJ. Core genome multilocus sequence typing scheme for stable, comparative analyses of *Campylobacter jejuni* and *C. coli* human disease isolates. J Clin Microbiol. 2017;55:2086–97. 28446571 10.1128/JCM.00080-17 PMC5483910

[20] Zielezinski A, Vinga S, Almeida J, Karlowski WM. Alignment-free sequence comparison: benefits, applications, and tools. Genome Biol. 2017;18:186. 28974235 10.1186/s13059-017-1319-7 PMC5627421

[21] Munck N, Njage PMK, Leekitcharoenphon P, Litrup E, Hald T. Application of whole-genome sequences and machine learning in source attribution of *Salmonella* Typhimurium. Risk Anal. 2020;40:1693. 32515055 10.1111/risa.13510 PMC7540586

[22] Smith HL, Biggs PJ, French NP, Smith ANH, Marshall JC. Lost in the Forest: encoding categorical variables and the absent levels problem. Data Min Knowl Disc. 2024;38:1889–908.

[23] Liao S-J (2020) Statistical modelling for zoonotic diseases : a thesis presented in partial fulfilment of the requirements for the degree of Doctor of Philosophy in Statistics at Massey University, Palmerston North, New Zealand. (Massey University).

[24] Havelaar AH, et al. Unravelling the reservoirs for colonisation of infants with *Campylobacter* spp. in rural Ethiopia: protocol for a longitudinal study during a global pandemic and political tensions. BMJ Open. 2022;12: e061311. 36198455 10.1136/bmjopen-2022-061311 PMC9535169

[25] Platts-Mills JA, et al. Detection of *Campylobacter* in stool and determination of significance by culture, enzyme immunoassay, and PCR in developing countries. J Clin Microbiol. 2014;52:1074–80. 24452175 10.1128/JCM.02935-13 PMC3993515

[26] Davedow T, et al. PulseNet international survey on the implementation of whole genome sequencing in low and middle-income countries for foodborne disease surveillance. Foodborne Pathog Dis. 2022. 10.1089/fpd.2021.0110. 35325576 10.1089/fpd.2021.0110 PMC10863729

[27] Bushnell B, Rood J, Singer E. BBMerge—accurate paired shotgun read merging via overlap. PLoS ONE. 2017;12: e0185056. 29073143 10.1371/journal.pone.0185056 PMC5657622

[28] BBDuk Guide. DOE Joint Genome Institute https://jgi.doe.gov/data-and-tools/software-tools/bbtools/bb-tools-user-guide/bbduk-guide/.

[29] Kokot M, Długosz M, Deorowicz S. KMC 3: counting and manipulating k-mer statistics. Bioinformatics. 2017;33:2759–61. 28472236 10.1093/bioinformatics/btx304

[30] Seemann, T. mlst. https://github.com/tseemann/mlst.

[31] Jolley KA, Maiden MC. BIGSdb: scalable analysis of bacterial genome variation at the population level. BMC Bioinformatics. 2010;11:595. 21143983 10.1186/1471-2105-11-595 PMC3004885

[32] Dingle KE, et al. Multilocus sequence typing system for *Campylobacter jejuni*. J Clin Microbiol. 2001;39:14–23. 11136741 10.1128/JCM.39.1.14-23.2001 PMC87672

[33] Zomer, A. aldertzomer/cgST. (2022).

[34] Zhou Z, et al. GrapeTree: visualization of core genomic relationships among 100,000 bacterial pathogens. Genome Res. 2018;28:1395–404. 30049790 10.1101/gr.232397.117 PMC6120633

[35] Anderson MJ (2017) Permutational multivariate analysis of variance (PERMANOVA). In: Wiley StatsRef: Statistics Reference Online 1–15 (John Wiley & Sons, Ltd). 10.1002/9781118445112.stat07841.

[36] Oksanen J et al. (2024) Vegan: community ecology package.

[37] R Core Team. R: a language and environment for statistical computing. (R Foundation for Statistical Computing, Vienna, Austria, 2023).

[38] Wright MN, Ziegler A. Ranger: a fast implementation of random forests for high dimensional data in C++ and R. J Stat Softw. 2017;77:1–17.

[39] Kuhn and Max. Building predictive models in R using the caret package. J Stat Softw. 2008;28:1–26.27774042

[40] Kursa MB, Rudnicki WR. Feature selection with the boruta package. J Stat Softw. 2010;36:1–13.

[41] Brinch ML, et al. Comparison of source attribution methodologies for human campylobacteriosis. Pathogens. 2023;12:786. 37375476 10.3390/pathogens12060786 PMC10303420

[42] Njage PMK, Leekitcharoenphon P, Hald T. Improving hazard characterization in microbial risk assessment using next generation sequencing data and machine learning: predicting clinical outcomes in shigatoxigenic *Escherichia coli*. Int J Food Microbiol. 2019;292:72–82. 30579059 10.1016/j.ijfoodmicro.2018.11.016

[43] Ogutu JO, Piepho H-P, Schulz-Streeck T. A comparison of random forests, boosting and support vector machines for genomic selection. BMC Proc. 2011;5:S11. 21624167 10.1186/1753-6561-5-S3-S11 PMC3103196

[44] Machado G, Mendoza MR, Corbellini LG. What variables are important in predicting bovine viral diarrhea virus? A Random Forest Approach Vet Res. 2015;46:85. 26208851 10.1186/s13567-015-0219-7 PMC4513962

[45] Poorrashidi M, Hitchcock M, Xu J. Meta-analyses of the global multilocus genotypes of the human pathogen *Campylobacter jejuni*. Genome. 2024;67:189–203. 38427983 10.1139/gen-2023-0041

[46] Mather AE, Gilmour MW, Reid SWJ, French NP. Foodborne bacterial pathogens: genome-based approaches for enduring and emerging threats in a complex and changing world. Nat Rev Microbiol. 2024. 10.1038/s41579-024-01051-z. 38789668 10.1038/s41579-024-01051-z

[47] Wallace RL, et al. *Campylobacter jejuni* ST50, a pathogen of global importance: a comparative genomic analysis of isolates from Australia, Europe and North America. Zoonoses Public Health. 2021;68:638–49. 34041858 10.1111/zph.12853

[48] Belina D, et al. Occurrence and diversity of *Campylobacter* species in diarrheic children and their exposure environments in Ethiopia. PLOS Glob Public Health. 2024;4:e0003885. 39471170 10.1371/journal.pgph.0003885 PMC11521251

[49] Jaakkonen A, Kivistö R, Aarnio M, Kalekivi J, Hakkinen M. Persistent contamination of raw milk by *Campylobacter jejuni* ST-883. PLoS ONE. 2020;15: e0231810. 32315369 10.1371/journal.pone.0231810 PMC7173850

[50] Joensen KG, et al. Whole-genome sequencing to detect numerous *Campylobacter jejuni* outbreaks and match patient isolates to sources, Denmark, 2015–2017. Emerg Infect Dis. 2020;26:523–32. 32091364 10.3201/eid2603.190947 PMC7045838

[51] Admasie A, et al. Genomic diversity of *Campylobacter jejuni* and Campylobacter coli isolated from the Ethiopian dairy supply chain. PLoS ONE. 2024;19: e0305581. 39159178 10.1371/journal.pone.0305581 PMC11332940

[52] French NP, et al. Population Structure and Antimicrobial Resistance in *Campylobacter jejuni* and *C. coli* Isolated from Humans with Diarrhea and from Poultry, East Africa. Emerg Infect Dis. 2024;30:2079–89. 39320160 10.3201/eid3010.231399 PMC11431929

[53] Bloomfield SJ, et al. Long-term colonization by *Campylobacter jejuni* within a human host: evolution, antimicrobial resistance, and adaptation. J Infect Dis. 2018;217:103–11. 10.1093/infdis/jix561 29099940

[54] Djeghout B, et al. Comparative genomics of *Campylobacter jejuni* from clinical campylobacteriosis stool specimens. Gut Pathogens. 2022;14:45. 36476389 10.1186/s13099-022-00520-1 PMC9727990

[55] Moxon ER, Murphy PA. *Haemophilus influenzae* bacteremia and meningitis resulting from survival of a single organism. Proc Natl Acad Sci. 1978;75:1534–6. 306628 10.1073/pnas.75.3.1534 PMC411507

[56] Schiaffino F, et al. The epidemiology and impact of persistent *Campylobacter* infections on childhood growth among children 0–24 months of age in resource-limited settings. eClinicalMedicine. 2024;76: 102841. 39380966 10.1016/j.eclinm.2024.102841 PMC11460251

[57] Chen D, Havelaar AH, Platts-Mills JA, Yang Y. Acquisition and clearance dynamics of *Campylobacter* spp. in children in low- and middle-income countries. Epidemics. 2024;46: 100749. 38367286 10.1016/j.epidem.2024.100749 PMC10944168

[58] Wang Y et al. Quantitative multi-pathway assessment of exposure to fecal contamination for infants in rural Ethiopia. 2024.08.29.24312786. 2024. Preprint at 10.1101/2024.08.29.24312786.

