# Supplementary material for: Transmission pathways of Campylobacter jejuni between humans and livestock in rural Ethiopia are highly complex and interdependent
Source: Gut Pathog. 2025 May 3;17:26. doi: 10.1186/s13099-025-00691-7 (PMC12049777; doi:10.1186/s13099-025-00691-7)
Supplement: Supplementary file 2 — Additional file 2 [file 13099_2025_691_MOESM2_ESM.docx]

## Supplementary tables

Table S1: Legacy MLST profiles of 11 novel sequence types

| ST | aspA | glnA | gltA | glyA | pgm | tkt | uncA |
| --- | --- | --- | --- | --- | --- | --- | --- |
| 14350 | 9 | 17 | 5 | 10 | 11 | 1164 | 3 |
| 14360 | 14 | 17 | 5 | 2 | 11 | 3 | 972 |
| 14365 | 188 | 313 | 876 | 2 | 89 | 5 | 6 |
| 14374 | 764 | 17 | 5 | 2 | 11 | 3 | 6 |
| 14388 | 765 | 34 | 27 | 33 | 1095 | 36 | 6 |
| 14396 | 2 | 55 | 2 | 3 | 2 | 1 | 5 |
| 14404 | 2 | 4 | 382 | 2 | 2 | 1 | 5 |
| 14407 | 4 | 7 | 10 | 4 | 90 | 7 | 1 |
| 14422 | 14 | 17 | 12 | 2 | 11 | 3 | 6 |
| 14423 | 9 | 17 | 52 | 10 | 10 | 3 | 5 |
| 14424 | 166 | 2 | 1 | 10 | 151 | 1 | 1 |

Table S2: 7-gene Sequence Types (ST) by source

|  | **Source** | | | | | | | |
| --- | --- | --- | --- | --- | --- | --- | --- | --- |
| **ST^*^** | **Infants** | **Siblings** | **Mothers** | **Chickens** | **Cattle** | **Goats** | **Sheep** | **Total** |
| **50** | 17 |  |  | 1 |  | 1 | 2 | 21 |
| **883** | 15 | 1 |  | 4 |  | 1 |  | 21 |
| **2042** | 18 |  |  |  |  | 2 |  | 20 |
| **19** | 8 | 2 |  | 7 |  | 1 |  | 18 |
| **849** | 7 |  | 3 | 6 |  |  |  | 16 |
| **2031** | 10 | 2 |  | 2 |  | 2 |  | 16 |
| **1038** | 4 | 2 |  | 5 | 2 | 1 |  | 14 |
| **305** | 8 |  |  | 3 | 1 | 1 |  | 13 |
| **362** | 10 |  |  | 2 |  |  |  | 12 |
| **1723** | 3 |  |  | 9 |  |  |  | 12 |
| **227** | 7 | 2 |  | 2 |  |  |  | 11 |
| **353** | 5 |  |  | 2 | 2 | 2 |  | 11 |
| **49** |  |  |  | 6 |  |  | 1 | 7 |
| **436** | 5 |  |  | 2 |  |  |  | 7 |
| **14423** | 6 |  |  | 1 |  |  |  | 7 |
| **1365** | 5 | 1 |  |  |  |  |  | 6 |
| **2100** | 1 |  |  | 5 |  |  |  | 6 |
| **2155** | 3 | 1 |  |  |  | 1 |  | 5 |
| **14365** | 1 |  |  | 2 | 1 |  | 1 | 5 |
| **14404** | 5 |  |  |  |  |  |  | 5 |
| **1751** | 4 |  |  |  |  |  |  | 4 |
| **3630** | 4 |  |  |  |  |  |  | 4 |
| **4624** | 4 |  |  |  |  |  |  | 4 |
| **10920** | 4 |  |  |  |  |  |  | 4 |
| **14424** | 4 |  |  |  |  |  |  | 4 |
| **403** |  | 3 |  |  |  |  |  | 3 |
| **824** | 3 |  |  |  |  |  |  | 3 |
| **14407** | 3 |  |  |  |  |  |  | 3 |
| **572** | 2 |  |  |  |  |  |  | 2 |
| **2304** |  | 2 |  |  |  |  |  | 2 |
| **5326** |  |  |  | 2 |  |  |  | 2 |
| **5596** | 2 |  |  |  |  |  |  | 2 |
| **14360** | 2 |  |  |  |  |  |  | 2 |
| **22** |  |  |  | 1 |  |  |  | 1 |
| **56** |  |  |  | 1 |  |  |  | 1 |
| **251** |  |  |  | 1 |  |  |  | 1 |
| **356** |  |  |  | 1 |  |  |  | 1 |
| **452** |  |  |  | 1 |  |  |  | 1 |
| **523** | 1 |  |  |  |  |  |  | 1 |
| **881** |  |  |  | 1 |  |  |  | 1 |
| **2109** |  |  |  | 1 |  |  |  | 1 |
| **3621** |  | 1 |  |  |  |  |  | 1 |
| **6839** | 1 |  |  |  |  |  |  | 1 |
| **14350** |  |  |  | 1 |  |  |  | 1 |
| **14374** |  |  |  | 1 |  |  |  | 1 |
| **14388** | 1 |  |  |  |  |  |  | 1 |
| **14396** | 1 |  |  |  |  |  |  | 1 |
| **14422** |  |  |  | 1 |  |  |  | 1 |
| **Total** | 174 | 17 | 3 | 71 | 6 | 12 | 4 | 287 |

^*^ Novel STs are highlighted in yellow

Table S3. PERMANOVA results

174 infant isolates

| Level A | Kebele | Kebele | Ganda | Infant |
| --- | --- | --- | --- | --- |
| Level B | Infant | Ganda | Infant | Sample |
| Sum of squares (%) | | | |  |
| A | 13.7 | 13.7 | 58.2 | 67.5 |
| Within A | 86.3^*^ | 86.3^•^ | 41.8 | 32.5 |
| B in A | 53.8^***^ | 46.7^***^ | 9.3^***^ | 22.5^**^ |
| Residual | 32.5 | 39.6 | 32.5 | 10.0^***^ |

^•^0.05≤p<0.10; ^*^0.01≤p<0.05; ^*^*0.001≤p<0.01; ^***^p<0.001

| Level A | Kebele | Kebele | Ganda | Infant |
| --- | --- | --- | --- | --- |
| Level B | Infant | Ganda | Infant | Sample |
| p-values | | | |  |
| Within A | 0.030^*^ | 0.053^•^ | 0.29 | 0.004^**^ |
| B in A | 0^***^ | 0^***^ | 0^***^ | 0^***^ |

97 infant isolates

| Level A | Kebele | Kebele | Ganda |
| --- | --- | --- | --- |
| Level B | Infant | Ganda | Infant |
| Sum of squares (%) | | | |
| A | 13.2 | 13.2 | 57.5 |
| Within A | 86.8^•^ | 86.8^•^ | 42.5 |
| B in A | 53.1^**^ | 46.8^***^ | 8.8 |
| Residual | 33.7 | 40.0 | 33.7 |

^•^0.05≤p<0.10; ^*^0.01≤p<0.05; ^*^*0.001≤p<0.01; ^***^p<0.001

| Level A | Kebele | Kebele | Ganda |
| --- | --- | --- | --- |
| Level B | Infant | Ganda | Infant |
| p-values | | | |
| Within A | 0.011^•^ | 0.017^•^ | 0.16 |
| B in A | 0.008^**^ | 9e-04^**^ | 0.20 |

Table S4. Valid accuracies for the model selection and *k*-mer model construction steps. The algorithm with the highest accuracy used for model construction is marked in bold. The valid accuracy is then given for the model with the algorithm that gave the highest accuracy.

|  | Model selection | | Model construction | | |  |
| --- | --- | --- | --- | --- | --- | --- |
|  | Average accuracy | |  |  |  | |
| Data set | Random forest | Logit boost | Selected algorithm | Valid accuracy (95% CI) | Kappa | |
| Dataset 1 | 0.937 | 0.944 | Random forest | 0.903 (0.829-0.953) | 0.87 | |
| Dataset 2 | 0.943 | 0.944 | Random forest | 0.961 (0.89-0.992) | 0.941 | |
